# Guiding the Ethics of Locator Devices in Dementia Care: Tutorial on Developing a Question-Based Ethical Framework

**DOI:** 10.2196/91667

**Published:** 2026-07-07

**Authors:** Jared Howes, Yvonne Denier, Tijs Vandemeulebroucke, Chris Gastmans

**Affiliations:** 1 Instituut voor Tropische Geneeskunde, Antwerp, Belgium; 2 Centre for Biomedical Ethics and Law, KU Leuven, Kapucijnenvoer 7 Blok g, Box 7001, Leuven, 3000, Belgium, 32 16 37 33

**Keywords:** dementia, health technology, cognition, wandering, ethics, bioethics

## Abstract

**Background:**

Locator devices are increasingly being used to help manage wandering among people living with dementia. While these technologies can be beneficial, they also raise significant ethical questions. These ethical questions are faced in both the development and use of these devices.

**Objective:**

The aim of this study is to develop and present a question-based ethical framework that can guide both the use and the development of locator devices in dementia care, ensuring that stakeholder needs and values are balanced effectively.

**Methods:**

A four-step process informed the framework’s creation: (1) literature collection (systematic searches of empirical, normative, and development-focused research were conducted); (2) content analysis (an inductive qualitative analysis of collected literature identified key ethical concerns, arguments, and stakeholder perspectives); (3) iterative framework drafting (insights were translated into open-ended questions and refined through regular team discussions); and (4) stakeholder consultation (feedback from health professionals, older adults, family members, developers, and ethicists was used to refine and finalize the framework).

**Results:**

The resulting framework is organized around 3 themes—vision, process, and impact—each containing guiding questions that encourage dialogical reflection and decision-making. For “use,” these questions help care communities clarify goals, define processes, and assess outcomes. For “development,” they aid developers in articulating device objectives, anticipating risks, and aligning product features with end-user values.

**Conclusions:**

By providing guiding questions that can be adapted to local needs and contexts, this framework fosters a more inclusive, transparent, and ethically accountable approach to developing and using locator devices in dementia care.

## Introduction

### Overview

Wandering is often perceived as one of the more challenging behavioral symptoms of dementia. Frequent wandering may lead to weight loss, sleep disturbances, fatigue, an increased risk of falls and fractures, early institutionalization, and elopement and getting lost, which carries a high risk of serious injury or death [1-3]. Although the negative aspects of wandering are most often discussed, its potential positive aspects are increasingly being recognized. For instance, wandering can reinforce habitual practices, involve beneficial physical activity, and be a form of exploration, particularly in new environments [4,5]. Furthermore, wandering may be a source of meaning to a person living with dementia, even if caregivers do not fully understand the motivations behind this behavior [6]. Freedom of movement is an important component of autonomy, valued by persons with dementia and their supporters alike [7]. Recognizing these positive aspects more deeply, the care response to wandering behavior has shifted from total prevention to risk mitigation and promotion of safer walking activities [8].

To address wandering in a way that supports autonomy and maintains safety, caregivers are increasingly turning to technology [9-11]. In particular, locator devices—also referred to as location tracking devices, electronic tracking devices, real-time location systems, monitors, personal safety alarms, or surveillance devices [12]—play an increasing role in wandering management, although current evidence for their effectiveness remains mixed [13]. Common devices include watches, bracelets, or fobs that use GPS, radio frequency, radio frequency identification, or Narrowband Internet of Things technology. Two primary features are common to most locator devices: real-time localization, which allows caregivers to determine the location of persons living with dementia, and passive monitoring, which provides alerts or alarms when specific events occur, such as crossing the boundaries of a geo-fenced “safe zone” or detecting a fall [12].

Within the normative and empirical literature, locator devices have primarily been examined from the perspective of their use, leaving the design and development processes comparatively underexplored. Both bodies of literature reflect this pattern. The normative literature has largely focused on the tension between promoting the autonomy of persons with dementia and maintaining their safety within the care environment [14,15]. Most ethical guidance focuses on the use of a particular technology in a particular care setting, such as radio frequency identification locator devices used within institutional care [16-18]. The empirical literature has likewise primarily focused on the perceptions and experiences of end users—persons with dementia, their spouses, family and friends, along with various professional caregivers [7]. Developers have only recently begun to be included in empirical research, mainly within studies exploring factors of locator device usability, acceptability, and adoptability [19-21].

A separate body of technical literature does address locator device development directly. Substantial work has been published detailing the design and development of locator technologies for dementia care [22,23]. However, much of this literature is concerned with technical and engineering challenges, and ethical considerations, when they appear, tend to be peripheral. A more ethically oriented strand has emerged from the broader dementia technology literature, including design guidelines [24], improvements to user-centered design [25,26], and efforts to address the challenges of fully involving persons living with dementia in development [27]. While this work explicitly engages with ethical dimensions, its focus remains methodological rather than constituting a dedicated ethical framework.

The importance of integrating ethics into the entire technology development process has long been recognized—development involves value-laden decisions that inevitably impact end users [28-30]. Developers of dementia technology share this concern, as reflected in the ongoing efforts to improve dementia product development and the experiences of locator device developers across both research and commercial settings [31]. Yet, despite this recognition, most dementia technologies are developed without explicit consideration for ethics [32]. While systematic evidence explaining this gap is lacking, plausible explanations include a combination of organizational pressures such as tight timelines and funding constraints [31,33], the absence of dedicated ethics expertise in smaller development teams that characterize much of the locator device industry [34], and, ultimately, a lack of accessible, practical ethics resources suited to the real constraints of development [35].

There is a clear need for an ethical framework that encourages explicit ethical consideration and supports developers in navigating ethical tensions. However, the complex diversity within the locator device domain makes developing such a framework challenging [36]. Development of locator devices is undertaken within commercial and university settings worldwide, involving teams ranging from individuals and small groups to small- to medium-sized organizations and multinational consortiums or corporations. Similarly, these devices are used by a diverse range of stakeholders, including informal and formal caregivers, home health aides, community volunteers, emergency services, and persons living with various stages of dementia [12]. The development and use of locator devices are shaped by stakeholders’ motivations, values, and priorities, as well as by broader contextual factors such as cultural values, legal regulations, health care organizations, and government support. As such, to meet the needs of developers and users, an ethical framework must be flexible enough to accommodate the diverse contexts of locator device development and use.

In response to this need, this paper introduces a question-based ethical framework to guide the development and use of locator devices in dementia care. A question-based approach has several advantages. Open-ended questions convey ethical content in a nonprescriptive way, encouraging adaptation to specific contexts. Because the questions can be revisited without following a fixed sequence, the framework can be integrated into ongoing ethical dialogue during active locator device development or use. The framework’s creation process, including the theoretical background and methods used, will be described prior to its introduction.

### Theoretical Background

This ethical framework is the result of a research project containing an empirical research line [12,31] and an applied ethics research line [15]. Developing this framework, therefore, requires integrating empirical data and normative analysis. Currently, there are no standard integration methods [37,38]. Indeed, while there are many methodologies, such as consultative, dialogical, or hermeneutical, the actual process of integrating empirical data and normative analysis remains vague even among experienced researchers [39]. At some point, a critical step must be taken that transcends methodological prescription or a priori explanation. What can be thoroughly outlined and explained, however, are the preconditions, tasks, and processes that form the groundwork for this step. Deliberately integrating strengths from various methods of bioethical inquiry improves this groundwork by enhancing the reflexivity, rigor, and transparency of the framework’s development process [40].

Prior to outlining our methods, we want to acknowledge the conceptual commitments shaping our approach. We have chosen to develop a framework intended to be used dialogically. This decision stems from the central role dialogue plays in both dementia care ethics and dementia technology development. In dementia care, the relational encounter between a vulnerable person and a caregiver forms the ethical basis of care—an encounter in which meaningful responses to vulnerability emerge from shared dialogue between persons with dementia (as far as their abilities allow), their families, professional caregivers, and others [41,42]. When a person with dementia begins to wander at night, a dialogue about how to respond will occur that involves the stakeholders’ values: the person’s expressed wishes, the family’s safety concerns, and the caregiver’s duty of care, for example. In dementia technology development, dialogue is equally central. Participatory design methods place the dialogical encounter between developers and end users, including persons with dementia, at the heart of creating more effective and ethically sound technologies [26,27,43,44]. For example, workshops that allow persons with dementia to experience potential design choices and express their opinions. Furthermore, our interview study with developers of locator devices found that engaging in dialogue with end users, domain experts, and peers was a central strategy for navigating ethical challenges in their work [31]. Therefore, working toward a framework rooted in dialogue would complement and strengthen the ethical practices that presently exist in dementia care and dementia technology development.

## Methods

### Overview

To ensure reflexivity and rigor, we developed a 4-step process that draws upon the strengths of different methods of bioethical inquiry. Each method typically requires lengthy explanation; however, due to space limitations, only key aspects will be reported. A complete accounting of the methods can be found in Multimedia Appendix 1.

### Step 1: Systematic Literature Collection

**Figure 1.**
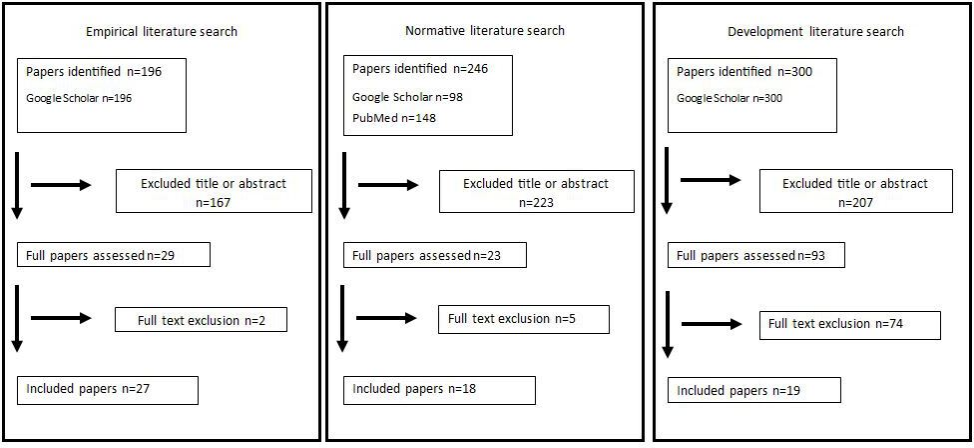
An overview of the systematic search process (adapted from Nicolini et al [47], which is published under Creative Commons Attribution 4.0 International License [48]).

The first step involved a systematic review of empirical and normative literature across 3 areas relevant to locator device development and use [45,46]. Using predefined inclusion and exclusion criteria (Multimedia Appendix 1), 64 papers were identified: empirical literature (n=27), covering stakeholder perceptions, implementation, and usability and acceptability; normative literature (n=18), focused on ethical arguments related to locator devices; and development literature (n=19), comprising guidelines, recommendations, and project outputs (Figure 1). This systematic approach enhances reflexivity and guards against overreliance on our own research.

### Step 2: Structuring and Integration of Literature

The second step involved the analysis of the previously gathered literature using a modified inductive qualitative analysis method [49]. The analysis ceased after finalization of codes rather than proceeding to the creation of an explanatory narrative structure. The analysis focused on identifying relevant elements from the gathered literature, such as arguments, insights, perspectives, concepts, recommendations, and themes, and organizing them into coded themes.

Each literature grouping underwent a 2-step coding process. First, in vivo coding was used to create an initial list of big picture concept codes. Second, through constant comparison, we reorganized these codes to identify subthemes. For example, within the empirical literature, all text fragments under the big picture concept “care goals” (identified during in vivo coding) were compared against one another, yielding subthemes such as “provide emotional support to persons with dementia,” “increase the safety of persons with dementia,” and “preserve beneficial aspects of wandering.” Next, the final codes from the 3 literature groups were compared, merged, and abstracted into a single list of ethical insights (Multimedia Appendix 2).

### Step 3: Development of Provisional Framework

The third step involved drafting a preliminary framework through iterative team discussion and revision, until the team reached agreement on a suitable version.

The ethical insights from step 2 were not reproduced directly in the framework; instead, they served as a reflective tool to identify potential blind spots, with their content synthesized into guiding questions tailored to use and development.

### Step 4: Stakeholder Consultation and Refinement

The fourth step entailed a feedback cycle to assess the preliminary framework’s relevance, clarity, and relatability across different stakeholder groups. Four stakeholder groups were consulted via convenience sampling, with deliberate effort to achieve diversity in sex, region, and professional background within each group (Table 1): technology developers, ethicists, health care professionals, and older adults, family members, and dementia patient advocates. This approach was chosen to maximize perspectives within the available time frame. Stakeholders were recruited through email, phone calls, and referrals. After giving informed consent, each participant received the provisional framework with instructions to provide critical feedback on its organization and content. Feedback was received in written and oral forms. Persons living with dementia or other cognitive impairments were not directly included in the feedback cycle due to time challenges, anticipated recruitment difficulties, and the barriers presented by a primarily written process. The feedback cycle was approved by the Social and Societal Ethics Committee of KU Leuven (file: G-2024‐7811-R2(MIN)). The cycle was initiated on January 7, 2025, and concluded on May 31, 2025. The feedback received was discussed within the research team and used to refine the final framework.

**Table 1.** Overview of stakeholder feedback participants.

Characteristics	Participants (N=17), n (%)
Sex	
Female	11 (64)
Male	6 (36)
Country	
Belgium	11 (64)
United States	2 (12)
Germany	2 (12)
Netherlands	1 (6)
New Zealand	1 (6)
Stakeholder group	
Technology developers	2 (12)
Professional ethicists	4 (24)
Health care professionals, caregivers	4 (24)
Older adults, family, patient advocates	7 (40)

## Results

### Framework for the Development and Use of Locator Devices in Dementia Care

The aim of this framework is to facilitate reflection and discussion to aid decision-making around ethical tensions in the development and use of locator devices in dementia care. It is intended for anyone involved in the development or use of locator devices in dementia care.

For users, this could include persons with dementia, families and friends, volunteer organizations, or professional care organizations, such as home health or institutional care facilities. In short, any informal or formal organization of persons devoted to responding to the vulnerabilities and needs of persons with dementia. For developers, this could include both technical and nontechnical professionals working in commercial or university development settings. For instance, engineers of various specializations, user experience and user interface researchers, product managers, marketers, sales and support, etc. This also includes academic researchers of all disciplines engaged in locator device development, whether working individually or as part of a consortium.

The framework has been organized to be inclusive, accommodating individuals with varying levels of expertise—from newcomers to seasoned professionals. To ensure accessibility, technical terms and jargon have been minimized. However, experienced readers will recognize concepts that are considered common knowledge within their respective disciplines.

Unlike conventional chronological ordering that begins with development, this framework begins with guiding ethical questions for the use of locator devices to underscore the importance of stakeholder-driven approaches in dementia care. How care communities want to conceive, integrate, and use locator devices within their care practices provides a robust foundation for considerations in development.

### How to Use These Guiding Questions

#### Overview

This framework includes 2 sections of guiding ethical questions, one for locator device use and the other for their development. Presenting them together allows for a more holistic view of the ethical issues involved. However, it is expected that persons will primarily use the section most relevant to their situation.

The questions are meant to be used within a deliberative dialogue—that is, a structured dialogue between individuals or groups that follows a formal process. While any opportunity for persons to gather and openly discuss, debate, and reflect upon these questions is valuable, a structured approach fosters greater consistency and depth in ethical reflection. These guiding questions aim to enhance, not replace, existing decision-making processes. No specific method or format is prescribed—cocreation, citizen juries, moderated meetings, and similar approaches are all compatible with this framework. However, any process must satisfy the following 5 conditions—derived from the accountability for reasonableness framework—which collectively define a deliberative dialogue [50,51].

The process should adhere to the following conditions:

- Inclusivity—engage as wide a group of stakeholders as possible and ensure their meaningful participation.
- Reasonableness—base decisions on reasonable evidence and arguments.
- Transparency—maintain as much transparency as feasible about the process, decisions, and their rationales to relevant stakeholders.
- Revisability—allow for revisions based on new evidence.
- Enforcement—ensure that the previous conditions are met through procedures and regulations.

What might this process look like in practice? Consider the following: volunteer organizations or charities (eg, Project Lifesaver or WanderSearch) are often the primary drivers of locator device use [52,53]. These groups work with local emergency services to provide resources, training, and devices for persons with dementia and their families. They could adopt this framework as part of their intake procedures for new clients. Adhering to the 5 conditions could involve a structured meeting process, ongoing dialogue among stakeholders, and accessible reports or meeting minutes shared with all participants. Whereas a small development team could embed these questions into regular internal and cross-team meetings, replacing vague prompts like “Does anyone have any questions or concerns?” with more targeted reflection that enhances team discussion and decision-making [54]. Families could set aside time to come together and work through the framework to better understand their own viewpoints, needs, and understanding regarding locator devices; and although these questions are designed to guide dialogue among people, they can also be used independently to clarify one’s own values.

#### Disclaimer

Several important considerations apply when using this framework. Not all questions will be relevant to every person or community. For instance, a person living with dementia and their spouse likely do not need to reflect on institutional policy within their own household, though they might wish to understand local care service policies. Accordingly, these questions are not exhaustive. Although relatively comprehensive, this list is not fixed. New questions, concerns, or topics may emerge that are not currently addressed. This framework should therefore be treated as a living document: one that can be revised, expanded upon, and adapted to meet the needs of specific persons and groups, provided it remains aligned with the 5 conditions of accountability for reasonableness. Finally, it is important to acknowledge what this framework can—and cannot—do. It can support deeper ethical reflection and help direct dialogue toward meaningful considerations. However, this framework cannot deliver definitive answers to ethical questions. It is not a flowchart that can be followed to a clear singular answer. An answer can only emerge from the shared dialogue between the individuals using this framework in particular circumstances.

The questions are organized around 3 main themes: vision, process, and impact. Each theme begins with a brief description, followed by key questions, some of which include subquestions to guide more detailed exploration of ethical issues.

### Use: Guiding Ethical Questions for Locator Device Use

#### Vision

A vision for the use of locator devices should be established before their implementation in care practices [18]. A clear vision clarifies the desired goals, the guiding values, and expectations for everyone involved in using locator devices [55]. A good vision supports alignment between stakeholders, helps set limits on how a device will be used, and orients how locator devices will fit within care practices.

Establishing a strong vision is important because it can be easy to blur or subtly shift who benefits from using a device, leading to scenarios where it is “not the person who is being monitored who benefits from the system, but rather the person doing the monitoring—the care organization, care professions, or informal carers” [25]. For instance, a common goal driving locator device use is to grant persons with dementia increased freedom of movement [56]. However, there is no guarantee that additional freedom will be given by a caregiver merely because a locator device is in use [13,57]. In such cases, the benefit is shifted entirely to caregivers, although persons with dementia still shoulder the burdens of device use. Textbox 1 contains a series of questions to help facilitate the clarification of vision on locator device use.

Textbox 1. **Guiding questions for the vision of locator device use.**

**What are our care goals in relation to using locator devices?**

- Where or with whom do we see the need for a locator device (eg, persons with dementia, caregivers, community, and institution)?
- What benefits do we seek for persons with dementia through locator device use? For caregivers? For other stakeholders?
- Which benefits are most important and should be prioritized?
- What reasons or motivations exist for and against using locator devices to achieve these benefits?

**What are the expectations for persons in relation to using locator devices?**

- Who are the relevant stakeholders connected to locator device use (eg, persons with dementia, caregivers, friends, technical staff, emergency services, and others)?
- Do we understand what locator devices can and cannot do (ie, strengths and limitations of the technology itself)? How can we make this information understandable for all stakeholders, including persons with dementia and their supporters?
- How do we picture using locator devices? For example, do we see the device as:
  - A safety net we activate only in emergencies?
  - A passive monitor that alerts us when it detects possible danger?
  - An active monitor that provides us continuous updates about the wearer?
  - A supportive tool that helps persons with dementia stay independent?
- When would it be appropriate and when would it be inappropriate to use a locator device (eg, when walking alone, when walking with a friend, and when showing high risk for wandering)?
- How would the use of locator devices fit into our current policy to managing challenging behavior such as wandering?

**What values are important in guiding our use of locator devices?**

- How might the values of autonomy, safety, dignity, trust, and transparency be relevant?
- What other values are relevant? And why?

#### Process

Processes and procedures need to be defined for the use of locator devices [58]. These measures are important for building trust in the technology and boosting user confidence [14,59]. When thoughtfully established, they prevent rushed, one-and-done, or all-or-nothing decisions. Good processes and procedures help caregivers, family, and others support a person living with dementia in making the best decisions possible, given their abilities.

Fluctuating decision-making capacity, for example, is a major challenge in every care context where locator devices might be used [15]. Persons with dementia who have decision-making capacity have the right to make an informed decision about whether to use locator devices [16]. However, dementia often causes fluctuations in capacity, meaning an individual’s ability to make specific decisions changes over time—sometimes even daily [15]. Thus, someone who can make an informed decision in the morning may no longer have the ability to do so by the evening. Although no consensus exists on how best to address this variability, a structured, shared decision-making process—initiated early and carried out over time—can help support persons living with dementia in making informed decisions [56,60,61]. Textbox 2 contains a series of guiding questions that can facilitate reflection on the processes and procedures related to locator device use.

Textbox 2. **Guiding questions for the process of locator device use.**

**What supporting infrastructure and resources are necessary to use locator devices in accordance with our ethical values?**

- What technical infrastructure and resources are needed to use and maintain locator devices? Do we have these?
- Do we have the necessary educational and support resources (eg, instructions, training sessions, tutorials, and troubleshooting) to train persons on how to use a locator device?

**How can we support and center persons with dementia throughout the decision-making process?**

- How can we structure the decision-making process to support shared decision-making between persons with dementia, their family and supporters, health care professionals, and other relevant stakeholders?
- How can we structure the decision-making process to ensure adequate time to reflect upon, try out (ie, trial), and adjust locator devices to the individual?
- How can we make key information (eg, benefits, risks, and limitations) about using locator devices accessible to all stakeholders, including persons with varying cognitive capacities?
- How can we accessibly explain what information is collected, who can see it, and how it is used by caregivers and potentially others (eg, scientific research and sold by a company)?

**How will we re-evaluate locator device use?**

- How will we re-evaluate to confirm our use is providing benefit to the person with dementia? To other stakeholders?
- How will we re-evaluate to confirm it is not causing negative impacts to the person with dementia (eg, altering behavior, social relations, and attitude)? To other stakeholders?
- How often should re-evaluations occur to ensure we respond promptly to changing circumstances?

**When using a locator device, how will we respond to a person with dementia, without decision-making capacity, who dissents or refuses to use the device?**

- How should our response be shaped by the nature of the dissent or refusal (eg, not wanting to be tracked, not wanting to wear or carry a device, and saying no to everything)?
- How should the frequency of dissent or refusal shape our response?
- Why is it necessary for a locator device to be used in this specific instance? For instance, is the device essential for health or physical safety and psychological comfort?
- What meaningful care alternatives to locator devices can we possibly offer?

#### Impact

Locator devices are ethically justified based on their ability to offer a proportional balance between benefits and harms in the least restrictive manner possible [15]. While this balancing often focuses on the potential impact to persons with dementia or caregivers, the broader levels in which locator device use is embedded and where ethical tensions arise also need to be considered (ie, micro, meso, and macro levels) [62,63]. This includes impacts to individuals and relationships, organizations and families (eg, family, or informal care network, home health organization, and care facilities), society (eg, city, region, and country), and the world (eg, cross-border collaborations and other nations).

These higher levels must be accounted for because locator devices rely on integration within organizations or communities to function. Caregivers, whether family and friends or professionals, must organize themselves to distribute roles and responsibilities necessary for a locator device to work well [64]. Consequently, while a locator device may provide benefits to a person with dementia, cumulative negative impacts on other levels may shift the balance of benefits and harms in such a way that locator devices are not justified. For example, caregivers overwhelmed by alerts and alarms may experience alarm fatigue, becoming desensitized and less alert, which undermines their caregiving [65,66]. This can negatively impact the organization’s care practices, which may increase burden on caregivers and cause harm to other residents—if someone must drop care tasks to respond to an alarm, for instance. Textbox 3 contains a series of guiding questions that can facilitate ethical reflection on the anticipated impacts of locator device use.

Textbox 3. **Guiding questions on anticipating the impacts of locator device use.**

**How might locator device use positively or negatively impact individuals and relationships?**

- How might the privacy of persons with dementia, caregivers, and other stakeholders be impacted (eg, reveal personal habits, prevent the need for physical monitoring, and increase or decrease)?
- How might self-perception of persons with dementia be impacted (eg, make them feel empowered and independent, more dependent and less like themselves, and stigmatized)?
- How might caregivers’ self-perception be impacted (eg, feel empowered and feel like surveillance agents)?
- How might the relationship between caregiver and person with dementia be impacted (eg, cause or reduce conflict and create opportunities for high-quality contact)?
- How might persons with dementia’s social relationships be impacted (eg, increase or reduce social isolation)?

**How might locator device use positively or negatively impact organizations and families?**

- How might the way a locator device functions (eg, frequency of alerts or alarms) impact caregivers in their daily tasks and practices? For example:
  - Could caregivers be overwhelmed by alarms and alerts?
  - Could a false sense of safety be created?
  - Could the device improve caregivers’ understanding of situations and help reduce burdens?
  - Could the device create moments of respite?
  - How might institutional rules and policies about using locator devices impact professional caregivers and their work? For example:
  - Could policies contribute to moral distress (eg, mandated alarm response time forces caregivers to drop other important tasks)?
  - Could policies create feelings of distrust between frontline caregivers and management (eg, management is “watching” workers)?
- Would using locator devices be financially sustainable, considering direct and indirect costs (eg, cost of device, subscriptions, maintenance, and infrastructure upgrades)? Why or why not?
- Would using locator devices be sustainable from a care practice perspective (ie, integrate well or require high organizational effort and resources for maintenance, daily upkeep, and training)? Why or why not?

**How might locator device use positively or negatively impact society and the world?**

- How would the use of locator devices contribute toward or take away from a dementia-friendly society (eg, harmful stereotypes and stigmatization)?
- How would the use of locator devices be sustainable from an environmental perspective (eg, does it align with organizational sustainability goals)? Why or why not?

### Development: Guiding Ethical Questions for Locator Device Development

#### Vision

The use of locator devices will embody differing visions of how to address the needs of persons with dementia and their caregivers. These visions emerge iteratively throughout development, shaped by the interplay of many factors, including developers’ own values and experiences, organizational missions, characteristics of particular care communities, the surrounding business environment, and the careful balancing of stakeholders’ differing needs [31].

Given the variability of these factors, a single, universal vision for locator devices in dementia care does not exist. Should locator devices strive to be as unobtrusive as possible, seamlessly blending into the background of life? Or should they take a more obtrusive form, ensuring that persons with dementia remain at the forefront of caregivers’ attention? The answer to such questions will be dependent on the specific context of development. Textbox 4 contains questions to reflect upon and clarify the vision underlying locator device development.

Textbox 4. **Guiding questions for the vision of locator device development.**

**What are the goals in relation to developing our locator device?**

- In what contexts or situations do we see the need for locator devices (eg, within the home and within an institution)?
- What specific challenges do we want to address by developing this locator device (eg, safety, situational awareness, and falls)?
- What benefits do we seek for persons with dementia (eg, self-efficacy, autonomy, peace of mind, and safety)? What benefits for caregivers? For other stakeholders?
- Which benefits are most important and should be prioritized?
- What beneficial aspects of wandering do we want to preserve or promote (eg, habitual practices, meaningful activity, exercise, and exploration)?

**Who is the locator device intended for, and who is expected to support its use?**

- Should it be used independently by persons with dementia with minimal support?
- Should it be used by caregivers in collaboration with persons with dementia?
- Should it be used by caregivers on persons with dementia without their direct participation?

**Within which care community or communities will our locator device be used (eg, family or friends, volunteer groups, emergency services, home health organizations, and care facilities)?**

- Why this community or communities and not others?

**What values are important in guiding our development of locator devices?**

- How might the values of autonomy, safety, dignity, trust, security, and transparency be relevant?
- What other values are relevant? Why these values and not others?

#### Process

The disciplines involved in development bring their own processes, procedures, and best practices to their work. Ethical responsibility, beyond matters of technical competency and adherence to regulations, often centers on the adequate inclusion of stakeholders—including persons with dementia—in development and the anticipation and mitigation of potential harms or opportunities for misuse introduced by locator devices [24,43,67,68].

Ethical reflection should extend beyond internal development methods to also include external-facing elements essential for the successful deployment of a locator device. For example, best practices and guidelines established by developers to train and educate end users also serve as an opportunity to promote ethical use. Textbox 5 contains questions to spur deeper ethical reflection concerning aspects related to the development process of locator devices.

Textbox 5. **Guiding questions for the process of locator device development.**

**How can we ensure that end users and stakeholders are meaningfully involved throughout the entirety of the development process?**

- How can we ensure daily users are involved?
- How can we adapt our processes to fully support the inclusion of persons with dementia with varying capabilities?
- How can we ensure that those who participate in development (eg, in ideation, testing, and trialing) have access to and can benefit from the final product?

**How can the software and hardware features be made simple and easy to use for persons with dementia? For caregivers? For other stakeholders?**

- How can we empower caregivers to adjust, tailor, and customize devices to respond to changing situations (eg, when cognitive or other impairments change)?
- How can we enable device features to be customizable and fine-tunable (eg, alarm sensitivity, alerts, lights, and sounds)?
- How can we provide information that end users can act upon with minimal processing (eg, monitoring, battery level, and device failure)?

**How can we provide end users with clear recommendations on how to use our locator device effectively and ethically?**

- What methods should be used to develop best practices for our device, and how can we incorporate end users and stakeholders?
- How can we clearly convey best practices to users (eg, through packaging, instructions, marketing, and educational materials)?
- How can we clearly convey what our locator device can and cannot do (ie, strengths and limitations of technology itself)?
- How can we re-evaluate how our locator device is used to improve and update our best practice recommendations?

**How will we provide comprehensive support throughout the locator device lifecycle, including setup, installation, education, training, technical support, and after-sales servicing?**

- How can we ensure that our educational and training materials are accessible to users with varying levels of technical literacy or cognitive abilities?
- How can we build users’ confidence in their ability to use our locator device?
- How can we build users’ confidence in our locator device’s reliability through education and training?

**How can we anticipate potential risks or harms related to our device?**

- How might our locator device be vulnerable to data breaches (eg, if a person with dementia accidentally discards or loses the device)?
- How could someone potentially misuse our locator device (eg, to restrain or pacify, socially control, surveil, or use a device in an improper manner)?

**How can we minimize these potential risks or harms without compromising the core user experience?**

- How could we minimize these risks through design (eg, privacy by design)?
- How could we minimize these risks through best practices?

#### Impact

The use of locator devices in dementia care is ethically justifiable if they deliver a balanced ratio of benefits to harm in the least restrictive manner possible. For developers who are attempting to craft a product capable of delivering this balance, a thorough reflection on the potential impacts not only on individual end users but also on the broader levels where locator devices are embedded and where ethical tensions arise is necessary (ie, micro, meso, and macro) [62,63]. In addition to impacts on the individuals and relationships, impacts on organizations and families (eg, families, home health organizations, and care facilities), society (eg, city, region, and country), and the world need to be considered (eg, cross-border collaborations, international business partnerships, and other nations). Textbox 6 contains questions centered on the impacts resulting from locator device development.

Textbox 6. **Guiding questions on anticipating the impact of locator device development.**

**What are the impacts on individuals and relationships?**

- How might using our locator device positively or negatively impact the privacy of persons with dementia, caregivers, and other stakeholders (eg, reveal personal habits and prevent the need for physical monitoring)?
- How might using our locator device impact how persons with dementia view themselves (eg, make them feel more empowered and independent or more dependent and less like themselves)?
- How might using our locator device impact how caregivers view themselves (eg, make them feel like good caregivers and feel like surveillance agents)?
- How might using our locator device positively or negatively impact the relationship between caregiver and person with dementia?
- How might using our locator device positively or negatively impact the social relations of persons with dementia (eg, increase or reduce social isolation)?

**What are the impacts on organizations or families?**

- How could using our locator device positively or negatively impact caregivers’ practices, routines, etc (eg, overwhelm caregivers with alarms and alerts, enhance caregivers’ situational awareness, and reduce caregiving burdens)?
- What will happen to end users if our company discontinues operations? How will they be able to continue using our locator device?
- Will using our locator device require irreversible changes to end-user infrastructure?

**What are the impacts on society and the world?**

- How does our work—product, services, marketing, support, etc—contribute toward or take away from a dementia-friendly society (eg, harmful stereotypes and stigmatization)?
- How might our locator device contribute to negative dementia stereotypes (childish qualities and pathology-centric) via the device form factor, operation, or marketing materials, etc?
- How do our choices of materials, manufacturing location, business plan, etc, align with environmental sustainability goals, commitments, and responsibilities?

## Discussion

### Principal Findings

This framework is the result of a larger project combining empirical-ethical and applied-ethics research [12,15,31]. The framework benefits significantly from the inclusion of insights drawn from interviews with developers of locator devices, a stakeholder group largely absent in previous ethical discussions. Our framework possesses 3 overall main strengths: the creation process, its structure, and its content.

The framework’s creation methodology addresses a common shortcoming in health care ethics guidelines: the vague and often undocumented processes behind the creation of ethical guidance and recommendations [69]. While foundational work, such as systematic literature reviews, quantitative and qualitative studies, and stakeholder consultation, is usually documented, the process of moving from this work to final guidance is rarely detailed [17,18,70]. This lack of clarity poses a significant methodological challenge, particularly for integrating empirical and normative insights. To address this challenge, we developed a transparent, well-documented, and rigorous methodology that drew upon established methods of bioethics.

A second key strength of the framework is its question-based structure. As previously noted, this structure reflects and builds upon the importance dialogue plays in both dementia care ethics [41,42] and the development of locator devices [31]. In addition, this structure is well-suited to address the sociomateriality of device development and use—the idea that technologies are socially produced, interpreted, and used [71]. Locator devices are developed through socially embedded practices, including team discussions and stakeholder inclusion [31]. Their use is similarly socially situated: identical devices can be used in radically different ways. As a result, intended benefits are not always realized. This is illustrated clearly in practice: a device intended to increase outdoor freedom for a person with dementia may fail to deliver this benefit if caregivers continue to impose movement restrictions regardless of the device’s presence—the technology alone cannot produce the desired outcome without corresponding changes in social practices and relationships [13,57]. Achieving benefits requires caregivers to organize themselves and adapt their practices accordingly. Thus, there is a need to better align the social (relationships, norms, and policies) and technical (functionality and infrastructure) dimensions of locator device development and use [64,72]. This alignment requires ethical reflection that does not separate a technology from its context of use [73].

Thus, sociomateriality demands flexibility and responsiveness to a particular care context. A question-based approach meets this demand due to its transferability [74]. By providing open-ended prompts to be discussed within specific care practices, it supports communities—families, volunteer groups, care institutions, and development teams—in reflecting deeper on the ethics of device development and use. Thus, a question-based structure supports ethical practice by facilitating the dialogue stakeholders need to ethically align social and technical elements within their care practices.

A third strength is that it broadens ethical reflection by adopting a relational and reflexive approach to the development and use of locator devices. Prior ethical analyses have often taken a narrow, individualistic perspective, focusing predominantly on the tension between autonomy and safety for persons with dementia [14,15]. However, a growing body of literature advocates for a more relational perspective, highlighting interdependencies between persons with dementia and caregivers [60]. Ethical reflection has thus expanded to include not only the best interests of persons with dementia but also their family members [17], potential negative impacts on caregivers—such as privacy concerns or alarm fatigue [14,16,75]—and negative effects on other residents within shared living environments [18]. Despite this relational shift, the existing scope remains relatively limited to caregivers and persons with dementia.

Our framework builds upon these relational insights, further expanding ethical considerations to encompass organizational, societal, and global concerns. By recognizing the broader network of relationships affected by these technologies, the framework provides a more comprehensive relational understanding of their development and use. Moreover, this broader reflection integrates both an ethics of carefulness and desirability [76]. Its open-ended questions regarding vision, process, and impact do more than simply help identify and mitigate potential harms. They actively encourage stakeholders to reflect critically on their motivations, biases, processes, and goals. Thus, this framework fosters thoughtful, self-aware decision-making grounded in ethical dialogue among stakeholders.

### Limitations

Our framework has several important limitations. First, a notable limitation is the absence of persons with dementia from the stakeholder consultation. This is a tension we acknowledge openly: a framework that asks caregivers and developers to center persons with dementia should itself model that commitment. Future iterations should prioritize their direct involvement through adapted formats (eg, verbal rather than written) better suited to their abilities and preferences consistent with the participatory design methods referenced throughout this paper. Second, as a question-based framework, it inherently offers “thin” normative guidance—invoking ethical concepts such as autonomy or safety without prescribing what they specifically mean or require in practice. This stands in contrast to a thicker approach that might stipulate, for instance, how autonomy should be weighed against safety in a particular care setting. While this supports transferability across diverse contexts, it means the framework does not provide definitive ethical answers. Finally, this work is situated predominantly within a Western, Educated, Industrialized, Rich, and Democratic context [77]. The project was conceptualized and conducted by American and Belgian researchers funded by the Flemish government, and both the primary empirical study and normative literature reflect predominantly Western perspectives. Consequently, despite the framework’s highly transferable design, its applicability beyond Western, Educated, Industrialized, Rich, and Democratic contexts may be limited. Future research should incorporate non-Western perspectives by, for instance, conducting a major round of feedback and revisions in an explicitly non-Western context to help adapt the framework to local needs and work to strengthen global approaches to bioethics. Additionally, empirical validation of the framework in practice represents an important next step. Specifically, future research should examine whether and how the framework facilitates meaningful ethical deliberation across diverse development and care contexts.

### Conclusions

This paper has presented a question-based ethical framework to help guide the development and use of locator devices in dementia care, created by integrating empirical and ethical insights through a structured, transparent process. This framework (1) supports transferability and context-specific action, (2) broadens ethical reflection, and (3) facilitates reflexivity. Consequently, this framework emphasizes stakeholder dialogue, openness to diverse contexts, and responsiveness to the sociomaterial dynamics of locator tracking device development and use. It is offered not as a finished product but as a starting point—one that invites critical engagement, adaptation, and refinement through the very kind of deliberative dialogue it is designed to support.

## Supplementary material

10.2196/91667 Multimedia Appendix 1 Overview of methods.

10.2196/91667 Multimedia Appendix 2 Overview of ethical insights derived from the synthesis of location tracking device literature (step 2).
