# Supplementary material for: Guiding the Ethics of Locator Devices in Dementia Care: Tutorial on Developing a Question-Based Ethical Framework
Source: J Med Internet Res. 2026 Jul 7;28:e91667. doi: 10.2196/91667 (PMC13340572; doi:10.2196/91667)
Supplement: Multimedia Appendix 1 [file jmir-v28-e91667-s001.docx]

The following supplemental material consists of an overview of the methods used in the development of the framework presented in “An Ethical Framework To Guide The Development And Use Of Location Tracking Devices In Dementia Care”. It details the methods used in the systematic search for and gathering of relevant literature (step 1), the analysis of this literature using inductive qualitative analysis (step 2), the development of the provisional framework (step 3), and the stakeholder consultation, revision, and finalization of the framework (step 4).

**Step 1 – Systematic literature collection**

The systematic search aimed to gather papers from three literature areas that focus on locator devices in dementia care: normative, empirical, and development. The included literature can be found at the end of this document. The inclusion and exclusion criteria were as follows:

## Inclusion and exclusion criteria

Normative literature was included if they met the following criteria.

| Inclusion criteria | Exclusion criteria |
| --- | --- |
| Publications that focus on locator tracking devices in dementia care. | Publications focusing on other technologies (e.g., alarms, electronic locks) |
| Normative publications as defined by Mertz (2019) as “literature that (i) aims to evaluate judgements, decisions, acts, (social) practices, technologies, institutions, organizations, general states of the world from a moral or legal point of view and/or to define/set out which decision or course of action is or should be morally or legally necessary, prohibited or permitted; or that (ii) aims to develop, interpret or criticize the evaluative or prescriptive concepts required for this” | Publications using an empirical design (i.e., quantitative or qualitative). |
| Language is English. | Non-English. |

Empirical literature was included if they met the following criteria.

| Inclusion criteria | Exclusion criteria |
| --- | --- |
| Publications that focus on the ethics of or implementation of locator tracking devices in dementia care. | Publications focusing on other technologies (e.g., alarms, electronic locks) |
| Publications using an empirical design (i.e., quantitative, qualitative, mixed methods). | Publications using non-empirical methods). |
| Language is English. | Non-English. |

Development literature was included if they met the following criteria.

| Inclusion criteria | Exclusion criteria |
| --- | --- |
| Publications that focus on locator tracking devices in dementia care. | Publications focusing on other technologies (e.g., alarms, electronic locks) |
| Publications that focus on design and development (e.g., participatory design, design recommendations and guidelines, technical memorandum). | Publications that focus on use |
| Language is English. | Non-English. |

## Search strings

Normative literature search strings

*Google Scholar:* (dementia or azheimer’s) AND ("wander") AND ("global positioning System" OR "locator" OR "tracker" OR "geofencing") AND ("ethics" OR "normative" OR "bioethics")

*Pubmed:* (aged[title/abstract] OR elder*[title/abstract] OR elder[title/abstract] OR “older persons”[title/abstract] OR “persons with dementia”[title/abstract] OR “people with dementia”[title/abstract] OR “patients with dementia”[title/abstract] OR dementia[title/abstract] OR Alzheimer[title/abstract] OR “Alzheimer’s disease”[title/abstract] OR geriatrics[title/abstract] OR “older adult”[title/abstract] OR “cognitive impairment”[title/abstract] OR ageing[title/abstract] OR psychogeriatrics[title/abstract] OR “cognitive disorders”[title/abstract] OR "Aged"[Mesh] OR "Dementia"[Mesh:NoExp] OR "Alzheimer Disease"[Mesh] OR "Dementia, Vascular"[Mesh:NoExp] OR "Geriatrics"[Mesh] OR "Cognitive Dysfunction"[Mesh] OR "Cognitive Aging"[Mesh] OR "Lewy Body Disease"[Mesh]) AND (“residential care”[title/abstract] OR wander*[title/abstract] OR wander[title/abstract] OR elopement[title/abstract] OR trailing[title/abstract] OR missing[title/abstract] OR lost[title/abstract] OR looping[title/abstract] OR “aimless walking"[title/abstract] OR sundowning[title/abstract] OR escape[title/abstract] OR restlessness[title/abstract] OR pacing[title/abstract] OR Wandering Behavior[Mesh]) AND (tracking device*[title/abstract] OR tracking[title/abstract] OR GPS[title/abstract] OR “global positioning system”[title/abstract] OR wearable*[title/abstract] OR “locator devices”[title/abstract] OR monitor*[title/abstract] OR tagging[title/abstract] OR “perimeter monitoring”[title/abstract] OR “assistive technology”[title/abstract] OR “app”[title/abstract] OR application[title/abstract] OR rfid[title/abstract] OR “electronic tracking”[title/abstract] OR “electronic tagging”[title/abstract] OR “electronically tagged”[title/abstract] OR “location surveillance”[title/abstract] OR “locator technology”[title/abstract] OR "wander management"[title/abstract] OR “surveillance technology”[title/abstract] OR "Wearable Electronic Devices"[Mesh]) AND (ethic*[title/abstract] OR ethical[title/abstract] OR concerns[title/abstract] OR ethics OR philosophy[title/abstract] OR philosoph*[title/abstract] OR moral*[title/abstract] OR reflection*[title/abstract] OR bioethics[title/abstract] OR “ethical analysis”[title/abstract] OR principle-based-ethic*[title/abstract] OR “medical ethics”[title/abstract] OR "Bioethical Issues"[Mesh] OR "Bioethics"[Mesh] OR "Ethical Theory"[Mesh] OR "Philosophy"[Mesh] OR “theology”[Mesh])

Empirical literature search string

*Google scholar:* (dementia or Alzheimer’s) AND ("wander") AND ("GPS" OR "locator" OR "tracker" OR "geofencing") AND "empirical" AND "qualitative"

Development Literature search string

*Google scholar:*  (dementia OR alzheimer's) AND ("wandering" OR "wander management") AND ("GPS" OR "location tracking" OR "geofencing") AND ("device design" OR "technology development" OR "wearable technology") AND ("study" OR "research" OR "article")

A Google Scholar search string was used for all three literature areas: normative, empirical, and development. Each search string was crafted and refined to return a useable number of results (e.g., 300 vs. 5000). Google Scholar was chosen for its indexation of a wide variety of literature across academic disciplines. An additional Pubmed search string was used for the normative literature search to take advantage of a previously completed systematic review of argument based ethics literature (Howes et al., 2021).

The Google Scholar searches were performed using the “Publish or Perish” computer software (Harzing, 2007) which allows Google Scholar search results to be collected. As the searches returned a reasonable number of results (e.g., 300 at most), they were able to be collected and downloaded.

The results for each literature area were imported into Endnote bibliographic computer software where they were screened based upon the inclusion and exclusion criteria. Screening in Endnote followed the method outlined in Bramer et al. (2017), which entails screening title and abstracts within the same step.

**Step 2 Structuring and integration of literature.**

The following material consists of an overview of the inductive qualitative analysis process used in step 2 of “An Ethical Framework To Guide The Development And Use Of Location Tracking Devices In Dementia Care” to structure and integrate the literature gathered in step 1.

The literature systematically gathered in step one was imported into the NVIVO qualitative analysis software. The categories of literature were maintained. That is, the 18 normative articles, 27 empirical articles, and 19 development articles, were stored in separate folders.

Each category of literature underwent the following process. First, the articles were read so they became familiar to [JH]. Next, a first round of in-vivo coding was completed that focused on identifying big-picture themes or concepts. For example, the need for locator devices to be user-friendly—a broad concept. Next, a second round of coding was completed where the initial codes were examined more closely to identify sub-categories. To build on the previous example, user-friendliness 🡪 software features (e.g., geo-fencing) need to be adjustable. These codes and sub-codes were then refined through an iterative return to the original documents to come to a final organization of codes.

At this stage, inductive qualitative data analysis typically aims to synthesize, interpret, and explain the codes in narrative format. In this project, however, this step is omitted, as the goal is to produce a list of codes useful that support reflection and development of the ethical content of our framework, rather than performing a full-fledged qualitative data analysis process. The final product of this process can be found in a table in supplemental material 2.

**Step 3 Development of provisional framework**

The ethical insights generated in step 2 were not directly reproduced in the framework’s content, rather, used as a reflective tool to identify potential blind spots, their content was synthesized and expressed in a series of guiding questions tailored to use and development. Throughout this process the research team met regularly to discuss and refine the framework until agreement on a provisional framework was achieved.

**Step 4 – Stakeholder consultation and refinement**

The fourth step entailed a feedback cycle to assess the preliminary framework’s relevance, clarity, and relatability across different stakeholder groups. Four stakeholder groups were consulted (Table 2): technology developers, ethicists, health care professionals, and older adults, family members, and dementia patient advocates. Due to time constraints, anticipated recruitment difficulties, and the challenges of a primarily written process, persons living with dementia or other cognitive impairments were not directly included in this feedback cycle.

Participants were recruited directly either via email or telephone call. Snowball recruitment was also used, where potential participants were asked to forward the framework and contact information to anyone they thought might be interested in participating. After giving their informed consent, each individual was given the provisional framework and provided with instructions to provide critical feedback on any aspect of its organization and content. Feedback was received in written and oral forms. Oral feedback occurred via Microsoft teams and were not recorded. Notes were taken by the interviewer (JH) and dispersed to the team for discussion.

The feedback cycle was approved by the Sociaal-Maatschappelijke Ethische Commissie of KU Leuven, File: G-2024-7811-R2(MIN). The cycle was initiated on 7 January 2025 and concluded on 31 May 2025. The feedback received was discussed within the research team and used to refine the final framework.

Results of step 1 – Systematic search

Empirical Literature

(Balsinha et al., 2022; Brookman et al., 2023; Cooper et al., 2021; Freiesleben et al., 2021; Gaggi et al., 2020; Gathercole et al., 2021; Greenhalgh et al., 2018; A Grigorovich et al., 2021; Gullslett et al., 2022; A Hall et al., 2019; Haslam-Larmer et al., 2024; Holbø et al., 2013; Juul et al., 2019; Köster, 2021; Larnyo et al., 2022; E. L. Mahoney & D. F. Mahoney, 2010; Monville et al., 2022; Müller et al., 2010; Neubauer et al., 2022; Sriram et al., 2021; Turner & Berridge, 2023; Vermeer et al., 2019; Wangmo et al., 2019; Wood, 2020; Wrede et al., 2023; Wrede et al., 2021)

Normative Literature

(Bantry-White, 2018; Berridge, 2018; Cahill et al., 2019; Cenci et al., 2023; A. Grigorovich et al., 2021; A. Hall et al., 2019; Howes et al., 2022; Howes et al., 2021; Koo & Vizer, 2019; Landau & Werner, 2012; Lodha & De Sousa, 2020; Morton et al., 2019; Neubauer et al., 2018; Nordgren, 2018; Shore, 2023; Teipel et al., 2018; Wayne, 2018; Young et al., 2018)

Development Literature

(Abbas & Michael, 2022; Branco et al., 2021; Cullen et al., 2022; Dhakal et al., 2019; Dröes et al., 2019; Hendriks et al., 2013; Interreg North-West Europe Certification-D 2023; Jacklin et al., 2020; Edward L Mahoney & Diane F Mahoney, 2010; Meiland et al., 2017; Møller, 2020; Müller et al., 2017; Robinson et al., 2009; Spilker & Norby, 2019; Teipel et al., 2016; Toso et al., 2023; Vermeer et al., 2020; Wan, 2017; Wan et al., 2016)

References

Abbas, R., & Michael, K. (2022). Co-designing location-based services for individuals living with dementia: An overview of present and future modes of operation. *IEEE Technology and Society Magazine*, *41*(2), 42-46.

Balsinha, C., Iliffe, S., Dias, S., Freitas, A., Barreiros, F. F., & Gonçalves-Pereira, M. (2022). Dementia and primary care teams: obstacles to the implementation of Portugal's Dementia Strategy. *Prim Health Care Res Dev*, *23*, e10. https://doi.org/10.1017/s1463423621000876

Bantry-White, E. (2018). Supporting ethical use of electronic monitoring for people living with dementia: Social work's role in assessment, decision-making, and review [Article]. *Journal of gerontological social work*, *61*(3), 261-279. https://doi.org/10.1080/01634372.2018.1433738

Berridge, C. (2018). Medicaid Becomes the First Third-Party Payer to Cover Passive Remote Monitoring for Home Care: Policy Analysis. *J Med Internet Res*, *20*(2), e66. <https://doi.org/10.2196/jmir.9650>

Bramer, W. M., Milic, J., & Mast, F. (2017). Reviewing retrieved references for inclusion in systematic reviews using endnote. (1558-9439 (Electronic)).

Branco, R. M., Hendriks, N., Lenaerts, L., & Wilkinson, A. (2021). The Challenges of Creating Design Requirements for Products for People with Dementia. In R. Brankaert, C. Raber, M. Houben, P. Malcolm, & J. Hannan (Eds.), *Dementia Lab 2021: Supporting Ability Through Design* (Vol. 2, pp. 15-25). Springer. https://doi.org/10.1007/978-3-030-70293-9_2

Brookman, R., Parker, S., Hoon, L., Ono, A., Fukayama, A., & ... (2023). *Technology for dementia care: what would good technology look like and do, from carers' perspectives?* Springer. https://doi.org/10.1186/s12877-023-04530-9

Cahill, J., Portales, R., McLoughin, S., Nagan, N., Henrichs, B., & Wetherall, S. (2019). IoT/Sensor-Based Infrastructures Promoting a Sense of Home, Independent Living, Comfort and Wellness. *Sensors (Basel)*, *19*(3). https://doi.org/10.3390/s19030485

Cenci, A., Ilskov, S. J., Andersen, N. S., & Chiarandini, M. (2023). The participatory value-sensitive design (VSD) of a mHealth app targeting citizens with dementia in a Danish municipality. *AI Ethics*, 1-27. https://doi.org/10.1007/s43681-023-00274-9

Cooper, J., Burrow, S., & Pusey, H. (2021). What are the perceptions of people living with dementia, family carers, professionals and other potential stakeholders to the use of global positioning systems to promote safer outdoor walking?: a qualitative literature review. *Disabil Rehabil Assist Technol*, *16*(6), 614-623. https://doi.org/10.1080/17483107.2019.1686074

Cullen, A., Mazhar, M., Smith, M., Lithander, F., & ... (2022). *Wearable and portable GPS solutions for monitoring mobility in dementia: a systematic review*. mdpi.com. https://www.mdpi.com/1424-8220/22/9/3336

Dhakal, A., Alsadoon, A., Prasad, P. W. C., Maag, A., Elchouemi, A., & Maung, W. (2019). Wearable Devices for Monitoring Dementia Sufferers: A Review and Framework for Discussion. *Proceedings of 2019 11th International Conference on Knowledge and Systems Engineering (Kse 2019)*, 300-306. https://doi.org/10.1109/kse.2019.8919373

Dröes, R.-M., Vermeer, Y., Libert, S., Gaber, S., Wallcook, S., Rai, H., Cavalcanti Barroso, A., van der Molen-van Santen, J., Mangiaracina, F., & Beentjes, K. (2019). *Best practice guidance: human interaction with technology in dementia: recommendations based on the research conducted in the Marie Sklodowska Curie International Training Network INDUCT*. https://www.dementiainduct.eu/guidance/

Freiesleben, S. D., Megges, H., Herrmann, C., Wessel, L., & Peters, O. (2021). Overcoming barriers to the adoption of locating technologies in dementia care: a multi-stakeholder focus group study. *BMC Geriatr*, *21*(1), 378. https://doi.org/10.1186/s12877-021-02323-6

Gaggi, O., Kolasinska, A., Palazzi, C., & ... (2020). *Safety first? users' perception of wearable sensor networks for aging*. Springer. https://doi.org/10.1007/s11036-019-01234-6

Gathercole, R., Bradley, R., Harper, E., Davies, L., Pank, L., Lam, N., Davies, A., Talbot, E., Hooper, E., Winson, R., Scutt, B., Montano, V. O., Nunn, S., Lavelle, G., Lariviere, M., Hirani, S., Brini, S., Bateman, A., Bentham, P.,…Howard, R. (2021). Assistive technology and telecare to maintain independent living at home for people with dementia: the ATTILA RCT. *Health Technol Assess*, *25*(19), 1-156. https://doi.org/10.3310/hta25190

Greenhalgh, T., Wherton, J., Papoutsi, C., Lynch, J., Hughes, G., A'Court, C., Hinder, S., Procter, R., & Shaw, S. (2018). Analysing the role of complexity in explaining the fortunes of technology programmes: empirical application of the NASSS framework. *BMC Med*, *16*(1), 66. https://doi.org/10.1186/s12916-018-1050-6

Grigorovich, A., Kulandaivelu, Y., Newman, K., & ... (2021). *Factors affecting the implementation, use, and adoption of real-time location system technology for persons living with cognitive disabilities in long-term care …*. jmir.org. https://www.jmir.org/2021/1/e22831/

Grigorovich, A., Kulandaivelu, Y., Newman, K., Bianchi, A., Khan, S. S., Iaboni, A., & McMurray, J. (2021). Factors Affecting the Implementation, Use, and Adoption of Real-Time Location System Technology for Persons Living With Cognitive Disabilities in Long-term Care Homes: Systematic Review. *J Med Internet Res*, *23*(1), e22831. https://doi.org/10.2196/22831

Gullslett, M. K., Nilsen, E. R., & Dugstad, J. (2022). Next of kin's experiences with and attitudes towards digital monitoring technology for ageing people with dementia in residential care facilities. A qualitative study based on the voices of next of kin and care providers. *Scand J Caring Sci*, *36*(4), 1094-1103. https://doi.org/10.1111/scs.13009

Hall, A., Brown Wilson, C., Stanmore, E., & Todd, C. (2019). Moving beyond 'safety' versus 'autonomy': a qualitative exploration of the ethics of using monitoring technologies in long-term dementia care. *BMC Geriatr*, *19*(1), 145. https://doi.org/10.1186/s12877-019-1155-6

Hall, A., Wilson, C. B., Stanmore, E., & Todd, C. (2019). *Moving beyond 'safety'versus 'autonomy': a qualitative exploration of the ethics of using monitoring technologies in long-term dementia care*. Springer. https://doi.org/10.1186/s12877-019-1155-6

Harzing, A. W. (2007). Publish or perish. In. http://www.harzing.com/pop.htm.

Haslam-Larmer, L., Grigorovich, A., Shum, L., Bianchi, A., Newman, K., Iaboni, A., & McMurray, J. (2024). Factors That Influence Successful Adoption of Real-Time Location Systems for Use in a Dementia Care Setting: Mixed Methods Study. *JMIR Aging*, *7*, e45978. https://doi.org/10.2196/45978

Hendriks, N., Truyen, F., & Duval, E. (2013). Designing with dementia: Guidelines for participatory design together with persons with dementia. Human-Computer Interaction–INTERACT 2013: 14th IFIP TC 13 International Conference, Cape Town, South Africa, September 2-6, 2013, Proceedings, Part I 14,

Holbø, K., Bøthun, S., & Dahl, Y. (2013). Safe walking technology for people with dementia: what do they want? *Proceedings of the 15th international acm …*. https://doi.org/10.1145/2513383.2513434

Howes, J., & Gastmans, C. (2021). Electronic tracking devices in dementia care: A systematic review of argument-based ethics literature. Archives of Gerontology and Geriatrics, 104419. https://doi.org/10.1016/j.archger.2021.104419

Howes, J., Denier, Y., & Gastmans, C. (2022). Electronic Tracking Devices for People With Dementia: Content Analysis of Company Websites. JMIR Aging, 5(4), e38865. https://doi.org/10.2196/38865

Howes, J., Denier, Y., Vandemeulebroucke, T., & Gastmans, C. (2024). The Ethics of Electronic Tracking Devices in Dementia Care: An Interview Study with Developers. Science and Engineering Ethics, 30(3), 17. https://doi.org/10.1007/s11948-024-00478-0

Interreg North-West Europe Certification-D (2023). Dementia Friendly Guidelines for SMES. https://vb.nweurope.eu/projects/project-search/certification-d-certification-of-technological-products-for-people-with-dementia-to-support-smes-in-innovation-and-business-growth/news/dementia-friendly-guidelines-for-smes/

Jacklin, K., Pitawanakwat, K., Blind, M., Lemieux, A. M., Sobol, A., & Warry, W. (2020). Peace of mind: A community-industry-academic partnership to adapt dementia technology for Anishinaabe communities on Manitoulin Island. *J Rehabil Assist Technol Eng*, *7*, 2055668320958327. https://doi.org/10.1177/2055668320958327

Juul, A., Wilding, R., & Baldassar, L. (2019). The Best Day of the Week: New Technology Enhancing Quality of Life in a Care Home. *Int J Environ Res Public Health*, *16*(6). https://doi.org/10.3390/ijerph16061000

Koo, B. M., & Vizer, L. M. (2019). Examining Mobile Technologies to Support Older Adults With Dementia Through the Lens of Personhood and Human Needs: Scoping Review. *JMIR Mhealth Uhealth*, *7*(11), e15122. https://doi.org/10.2196/15122

Köster, A. (2021). *Determinants of User Acceptance of In-Home Monitoring Technologies for Persons with Dementia (PwD)*. essay.utwente.nl. http://essay.utwente.nl/87393/

Landau, R., & Werner, S. (2012). Ethical aspects of using GPS for tracking people with dementia: Recommendations for practice [Article]. *International Psychogeriatrics*, *24*(3), 358-366. https://doi.org/10.1017/S1041610211001888

Larnyo, E., Dai, B., Larnyo, A., Nutakor, J., & ... (2022). *Impact of actual use behavior of healthcare wearable devices on quality of life: a cross-sectional survey of people with dementia and their caregivers in Ghana*. mdpi.com. https://www.mdpi.com/2227-9032/10/2/275

Lodha, P., & De Sousa, A. (2020). Ethics of electronic tagging of dementia patients. *Indian J Med Ethics*, *V*(1), 83-84. https://doi.org/10.20529/ijme.2019.078

Mahoney, E. L., & Mahoney, D. F. (2010). Acceptance of wearable technology by people with Alzheimer's disease: issues and accommodations. *Am J Alzheimers Dis Other Demen*, *25*(6), 527-531. https://doi.org/10.1177/1533317510376944

Mahoney, E. L., & Mahoney, D. F. (2010). Acceptance of wearable technology by people with Alzheimer’s disease: issues and accommodations. *American Journal of Alzheimer's Disease & Other Dementias*, *25*(6), 527-531. https://doi.org/10.1177/1533317510376944

Meiland, F., Innes, A., Mountain, G., Robinson, L., van der Roest, H., García-Casal, J. A., Gove, D., Thyrian, J. R., Evans, S., & Dröes, R.-M. (2017). Technologies to support community-dwelling persons with dementia: a position paper on issues regarding development, usability, effectiveness and cost-effectiveness, deployment, and ethics. *JMIR rehabilitation and assistive technologies*, *4*(1), e6376.

Møller, A. (2020). The design of electronic tagging and tracking solutions to improve the safety and person-centered care for people with dementia. *Human Aspects of IT for the Aged Population. Healthy …*. https://doi.org/10.1007/978-3-030-50249-2_11

Monville, M., Schlögl, S., Weichelt, R., & ... (2022). Perspectives on Technology Use in Dementia Care–An Exploratory Study of Nursing Homes in Luxembourg. *… Conference on ICT for …*. https://doi.org/10.1007/978-3-031-29548-5_5

Morton, T., Atkinson, T., Brooker, D., Wong, G., Evans, S., & Kennard, C. (2019). Sustainability of community-based interventions for people affected by dementia: a protocol for the SCI-Dem realist review. *BMJ Open*, *9*(7), e032109. https://doi.org/10.1136/bmjopen-2019-032109

Müller, C., Wan, L., & Hrg, D. (2010). Dealing with wandering: a case study on caregivers' attitudes towards privacy and autonomy when reflecting the use of LBS. *Proceedings of the 2010 ACM International …*. https://doi.org/10.1145/1880071.1880082

Müller, I., Mertin, M., & Rolf, M. (2017). *Technology as an area of conflict between autonomy and safety-acceptance and attitudes of family caregivers in regard to technical assistance to ensure safe areas of movement for people with dementia diseases* International Conference on Information and Communication Technologies for Ageing Well and e-Health., https://www.scitepress.org/Papers/2017/62830/

Neubauer, N., Spenrath, C., Philip, S., Daum, C., Liu, L., & Miguel-Cruz, A. (2022). Identifying adoption and usability factors of locator devices for persons living with dementia. *Dementia (London)*, *21*(3), 862-881. https://doi.org/10.1177/14713012211065381

Neubauer, N. A., Lapierre, N., Ríos-Rincón, A., Miguel-Cruz, A., Rousseau, J., & Liu, L. (2018). What do we know about technologies for dementia-related wandering? A scoping review: Examen de la portée : Que savons-nous à propos des technologies de gestion de l'errance liée à la démence? *Can J Occup Ther*, *85*(3), 196-208. https://doi.org/10.1177/0008417418777530

Nordgren, A. (2018). How to respond to resistiveness towards assistive technologies among persons with dementia. *Medicine Health Care and Philosophy*, *21*(3), 411-421. https://doi.org/10.1007/s11019-017-9816-8

Robinson, L., Brittain, K., Lindsay, S., Jackson, D., Olivier, P., Robinson, L., Brittain, K., Lindsay, S., Jackson, D., & Olivier, P. (2009). Keeping In Touch Everyday (KITE) project: developing assistive technologies with people with dementia and their carers to promote independence. *International Psychogeriatrics*, *21*(3), 494-502. https://doi.org/10.1017/S1041610209008448

Shore, K. (2023). *Exploring the Care-Control Nexus Through Police Monitoring of Vulnerable Groups: A Case Study of Project Lifesaver*. uwspace.uwaterloo.ca. https://uwspace.uwaterloo.ca/handle/10012/20159

Spilker, H. S., & Norby, M. K. (2019). Understanding the role of technology in care: the implementation of GPS-technology in dementia treatment. *Ageing international*, *44*, 283-299. https://doi.org/10.1007/s12126-018-9340-z

Sriram, V., Jenkinson, C., & Peters, M. (2021). Carers using assistive technology in dementia care: an explanatory sequential mixed methods study. *medRxiv*. https://doi.org/10.1101/2021.04.08.21255110.abstract

Teipel, S., Babiloni, C., Hoey, J., Kaye, J., Kirste, T., & ... (2016). *Information and communication technology solutions for outdoor navigation in dementia*. Elsevier. https://www.sciencedirect.com/science/article/pii/S1552526015030289

Teipel, S., König, A., Hoey, J., Kaye, J., Krüger, F., Robillard, J. M., Kirste, T., & Babiloni, C. (2018). Use of nonintrusive sensor-based information and communication technology for real-world evidence for clinical trials in dementia. *Alzheimers Dement*, *14*(9), 1216-1231. https://doi.org/10.1016/j.jalz.2018.05.003

Toso, F., Brankaert, R., Hendriks, N., Lenaerts, L., & Wilkinson, A. (2023). Reflecting on Living Labs as Multi-Stakeholder Collaborative Networks to Evaluate Technological Products for People Living with Dementia. *International Journal of Environmental Research and Public Health*, *20*(3).

Turner, N. R., & Berridge, C. (2023). How I want technology used in my care: Learning from documented choices of people living with dementia using a dyadic decision making tool. *Inform Health Soc Care*, *48*(4), 387-401. https://doi.org/10.1080/17538157.2023.2252066

Vermeer, Y., Higgs, P., & Charlesworth, G. (2019). What do we require from surveillance technology? A review of the needs of people with dementia and informal caregivers. *J Rehabil Assist Technol Eng*, *6*, 2055668319869517. https://doi.org/10.1177/2055668319869517

Vermeer, Y., Higgs, P., & Charlesworth, G. (2020). Selling surveillance technology: semiotic themes in advertisements for ageing in place with dementia. *Social Semiotics*, *32*(3), 400-421. https://doi.org/10.1080/10350330.2020.1767399

Wan, L. (2017). *Dealing with wandering in dementia care: a developmental story of designing a GPS monitoring system and its challenges in a wider context*. 141.99.19.133. http://141.99.19.133/handle/ubsi/1440

Wan, L., Müller, C., Randall, D., & Wulf, V. (2016). Design of A GPS Monitoring System for Dementia Care and its Challenges in Academia-Industry Project. *ACM Transactions on Computer-Human Interaction*, *23*(5), 1-36. https://doi.org/10.1145/2963095

Wangmo, T., Lipps, M., Kressig, R. W., & Ienca, M. (2019). Ethical concerns with the use of intelligent assistive technology: findings from a qualitative study with professional stakeholders. *BMC Med Ethics*, *20*(1), 98. https://doi.org/10.1186/s12910-019-0437-z

Wayne, K. (2018). How Can Ethics Support Innovative Health Care for an Aging Population? *Ethics & Behavior*, *29*(3), 227-253. https://doi.org/10.1080/10508422.2018.1526087

Wood, E. (2020). *The Lived Experience of Using Safer Walking Technology: Supporting Meaningful Occupation and Identity for People with Early Stage Dementia*. pure.coventry.ac.uk. https://pure.coventry.ac.uk/ws/portalfiles/portal/53078081/EW_Thesis_Redacted.pdf

Wrede, C., Braakman-Jansen, A., & ... (2023). *Understanding acceptance of contactless monitoring technology in home-based dementia care: a cross-sectional survey study among informal caregivers*. frontiersin.org. https://doi.org/10.3389/fdgth.2023.1257009

Wrede, C., Braakman-Jansen, A., & van Gemert-Pijnen, L. (2021). Requirements for Unobtrusive Monitoring to Support Home-Based Dementia Care: Qualitative Study Among Formal and Informal Caregivers. *JMIR Aging*, *4*(2), e26875. https://doi.org/10.2196/26875

Young, Y., Papenkov, M., & Nakashima, T. (2018). Who Is Responsible? A Man With Dementia Wanders From Home, Is Hit by a Train, and Dies. *J Am Med Dir Assoc*, *19*(7), 563-567. https://doi.org/10.1016/j.jamda.2018.02.006
