# Supplementary material for: Guiding the Ethics of Locator Devices in Dementia Care: Tutorial on Developing a Question-Based Ethical Framework
Source: J Med Internet Res. 2026 Jul 7;28:e91667. doi: 10.2196/91667 (PMC13340572; doi:10.2196/91667)
Supplement: Multimedia Appendix 2 [file jmir-v28-e91667-s002.docx]

| **Table S1.** Overview of ethical insights derived from the synthesis of location tracking device literature (step 2). | |
| --- | --- |
| **Motivation/Purpose for LTD* use** | **Supporting Articles** |
| LTD is the best option among care alternatives. | (Landau et al., 2010; Niemeijer et al., 2011; Niemeijer et al., 2010; Sundgren et al., 2019; White & Montgomery, 2014; White et al., 2010; Wrede et al., 2021) |
| To facilitate/aid caregivers in providing higher quality care to persons with dementia. | (Felber et al., 2023; Freiesleben et al., 2021; Hall et al., 2017; Howes et al., 2024; Niemeijer et al., 2010; Sundgren et al., 2019; Wan, 2017; Wrede et al., 2021) |
| Out of Fear: To preserve institutional/personal reputation in response to societal pressures. | (Hall et al., 2017; Müller et al., 2010; Niemeijer et al., 2010; Wherton et al., 2019) |
| Out of obligation: LTD preserves safety of persons with dementia, therefore we must use it. | (Freiesleben et al., 2021; Hall et al., 2017; Landau et al., 2010; Landau et al., 2009; White et al., 2010) |
| **Care goals of LTD use** | **Supporting Articles** |
| Provide cognitive and emotional support to persons with dementia. | (Cooper et al., 2019; Freiesleben et al., 2021; Gullslett et al., 2021; Landau et al., 2010; Monville et al., 2022; Niemeijer et al., 2015; White & Montgomery, 2014; White et al., 2010; Williamson et al., 2017) |
| Preserve and support the autonomy and independence of persons with dementia. | (Bantry-White, 2018; Freiesleben et al., 2021; Gullslett et al., 2021; Hall et al., 2017; Howes & Gastmans, 2021; Landau et al., 2010; Landau & Werner, 2012; Liu et al., 2022; Monville et al., 2022; Niemeijer & Hertogh, 2008; Niemeijer et al., 2015; Remmers, 2010; Robinson et al., 2009; Welsh et al., 2003; Wherton et al., 2019; White & Montgomery, 2014; White et al., 2010; Wrede et al., 2021; Yang & Kels, 2016) |
| Enhance the safety of persons with dementia. | (Branco et al., 2021; Cooper et al., 2019; Freiesleben et al., 2021; Gullslett et al., 2021; Hall et al., 2017; Howes & Gastmans, 2021; Landau et al., 2010; Niemeijer et al., 2011; Olsson et al., 2013; White & Montgomery, 2014; Williamson et al., 2017) |
| Enhance the privacy of persons with dementia. | (Freiesleben et al., 2021; Gullslett et al., 2021; Hall et al., 2017; Howes & Gastmans, 2021; Niemeijer et al., 2015; Niemeijer et al., 2011; Niemeijer et al., 2010; Sundgren et al., 2019; Wan et al., 2016; Wrede et al., 2021; Wykes & Schueller, 2019) |
| *Location tracking device |  |
| Preserve and promote beneficial aspects of wandering. | (Müller et al., 2017; Niemeijer et al., 2015; Wherton et al., 2019)m |
| Support caregivers by providing peace of mind, empowering them, enhancing caregiving abilities. | (Berridge et al., 2021; Cooper et al., 2019; Felber et al., 2023; Freiesleben et al., 2021; Gullslett et al., 2021; Howes et al., 2024; Landau et al., 2010; Landau & Werner, 2012; Landau et al., 2009; Olsson et al., 2013; White et al., 2010; Williamson et al., 2017; Wrede et al., 2021) |
| Bolstering institutional security and functional efficiency. | (Niemeijer et al., 2010; Sundgren et al., 2019) |
| Reduce institutional risk and staff burden. | (Howes et al., 2022; Howes et al., 2024; Niemeijer et al., 2010) |
| **Evaluating need for LTD** | **Supporting Articles** |
| Where does the need for LTDs arise (e.g., with PwD, caregivers)? | (Niemeijer et al., 2010; Sundgren et al., 2019) |
| A clear risk model is needed to enable proactive and not reactive wander management strategies. | (Neubauer & Liu, 2021) |
| **Conceptualization of LTD as care intervention** | **Supporting Articles** |
| LTD use should have a defined procedure. | (Niemeijer et al., 2011) |
| LTD use should be beneficial and tailored to individual residents’ preferences, rituals, and daily structure (no blanket use). | (Dröes et al., 2019; Jacklin et al., 2020; Köhler et al., 2022; Møller, 2020; Niemeijer et al., 2011; Niemeijer et al., 2010; Tetley et al., 2017; Wan, 2017) |
| LTDs are best used to manage select situations (e.g., leaving safe zone/geo-fence) rather than constantly monitor. | (Møller, 2020; Wan, 2017) |
| LTDs as a primary intervention (i.e., no a-priori restrictions in place prior to LTD introduction). | (White et al., 2010) |
| LTDs as a secondary intervention (e.g., a back-up to another intervention method, such as a 'safety net' for constant monitoring). | (White et al., 2010) |
| LTDs as part of a multi-pronged approach involving high, low, and no technology strategies. | (Brittain et al., 2017; Neubauer et al., 2018) |
| LTD use viewed as form of restraint. | (Niemeijer et al., 2010) |
| LTD use viewed as form of Risk reduction | (Freiesleben et al., 2021; White et al., 2010) |
| LTDs used by, with, or on PwD? | (Branco et al., 2021; Gibson et al., 2016) |
| **LTD scope of use** | **Supporting Articles** |
| LTD is not and should not be a replacement for human contact or care. | (Gullslett et al., 2021; Howes & Gastmans, 2021; Møller, 2020; Niemeijer et al., 2010; Spilker & Norby, 2019; Sundgren et al., 2019; White & Montgomery, 2014; White et al., 2010; Wrede et al., 2021) |
| LTD is not suitable for all dementia phases. | (Landau et al., 2010) |
| LTDs not essential for health (as opposed to conventional care, like showers). | (Nordgren, 2018) |
| No guarantee LTD will increase safety. | (Howes & Gastmans, 2021; Sundgren et al., 2019) |
| The device physical form and software should be easy and simple to use, repair, update, and refurbish. | (Bastoni et al., 2021; Cooper et al., 2019; Interreg North-West Europe Certification-D 2023; Spilker & Norby, 2019; White et al., 2010) |
| The device physical form should be customizable and personalize to a PwD. | (Howes et al., 2022; Interreg North-West Europe Certification-D 2023; Wan, 2017) |
| Device software features and user interface characteristics should be customizable and enable choice and control. | (Berridge et al., 2021; Interreg North-West Europe Certification-D 2023; Møller, 2020; Wan, 2017; Wan et al., 2016) |
| LTDs should provide actionable information that requires minimal processing. | (Felber et al., 2023; Freiesleben et al., 2021; Hall et al., 2017; Wrede et al., 2021) |
| **LTD complementarity** | **Supporting Articles** |
| LTD should support the community it is embedded within through facilitating communication, coordination, and negotiation between community members (e.g., PWD, caregivers, family, friends, staff, etc.). | (Wan, 2017; Wan et al., 2016) |
| LTD should complement local wandering policy. | (Neubauer & Liu, 2021) |
| Device daily use and maintanence must support and compliment the established routines of family and institutional life (i.e., fit into their care and work practices). | (Branco et al., 2021) |
| **Impact of LTDs** | **Supporting Articles** |
| Potential for LTDs to disrupt care practices, avoid alarm/information overload, and minimize introducing new burdens (maintanence). | (Berridge et al., 2021; Grigorovich & Kontos, 2020; Howes & Gastmans, 2021; Niemeijer et al., 2010) |
| LTDs may create false sense of safety or security. | (Howes & Gastmans, 2021; Niemeijer et al., 2010) |
| LTDs violate privacy to achieve goods/benefits, and these should be directly benefiting to PwD. | (Cooper et al., 2019; Landau & Werner, 2012; Niemeijer et al., 2010; Sundgren et al., 2019) |
| LTD use may stigmatize/dehumanize PwD. | (Freiesleben et al., 2021; Howes & Gastmans, 2021; Landau et al., 2010; Niemeijer et al., 2015; White & Montgomery, 2014; Wrede et al., 2021) |
| Avoid exacerbating power asymmetries and existing marginalization of PwDs. | (Abbas & Michael, 2022) |
| Does device introduce inequality (e.g., between residents)? | (Niemeijer et al., 2015) |
| Avoid stigmatizing qualities in device and marketing (e.g., childish qualities). | (Dröes et al., 2019; Interreg North-West Europe Certification-D 2023; Mahoney & Mahoney, 2010) |
| **Decision making - process** | **Supporting Articles** |
| Informed consent required before tech use on/with PWD. | (Hendriks et al., 2013; Howes & Gastmans, 2021; Landau & Werner, 2012; Niemeijer et al., 2010) |
| Respect PwDs right to informed refusal. | (Grigorovich & Kontos, 2020) |
| No coerced use of LTDs. | (Howes & Gastmans, 2021; Landau et al., 2010; Niemeijer et al., 2010) |
| Decision making process should be started early, be structured, and be a shared decision-making process. | (Landau et al., 2010; Landau et al., 2011; Landau & Werner, 2012; Liu et al., 2022; Niemeijer et al., 2010; Sundgren et al., 2019; White & Montgomery, 2014; Wrede et al., 2021) |
| Proxy-decision making if PWD does not have capacity. | (Howes & Gastmans, 2021; Landau & Werner, 2012) |
| PwD Cooperation and assent should be sought. | (Cooper et al., 2019; Landau et al., 2010; White & Montgomery, 2014) |
| The interests of PwD should be prioritized. | (Niemeijer et al., 2010) |
| Need to balance interest of PwD and their family/caregivers. | (Landau & Werner, 2012) |
| Process should unfold over time and be tailored presentation of device to PwDs' particular histories, values, wants. | (Berridge et al., 2021; Cooper et al., 2019; White & Montgomery, 2014; White et al., 2010) |
| **Challenges in decision-making** | **Supporting Articles** |
| Fluctuating capacity challenges the informed consent/decision-making process | (Howes & Gastmans, 2021; Niemeijer et al., 2010; Sundgren et al., 2019) |
| Some professionals may not want to be involved in decision making on whether to use LTD, may doubt their expertise to sway/influence (or make) decision. | (Landau et al., 2010; Müller et al., 2010) |
| **Re-evaluation of an evolving practice** | **Supporting Articles** |
| Re-evaluate the benefits of using an LTD and allow for their withdrawal if non-beneficial. | (Berridge et al., 2021; Hall et al., 2017; Wan et al., 2016; Wykes & Schueller, 2019) |
| Re-evaluate whether LTD use causes negative change in PwD. | (Grigorovich & Kontos, 2020) |
| Re-evaluate LTD care policy impacts on caregiver practices. | (Brittain et al., 2017; Hall et al., 2019) |
| Empower continuous adaption and personalize LTDs to respond to changing caregiving needs. | (Gibson et al., 2019; Gibson et al., 2016; Hall et al., 2019; Spilker & Norby, 2019) |
| **Data and privacy** | **Supporting Articles** |
| Collected personal and contextual information should be kept confidential and protected by design. | (Berridge et al., 2021; Grigorovich & Kontos, 2020; Interreg North-West Europe Certification-D 2023; Wykes & Schueller, 2019) |
| Collect only the minimal necessary data (i.e., principle of data parsimony). | (Köhler et al., 2022) |
| Ensure transparency regarding collected data. Where are the data going? Who can use the data? Is the data being monetized? | (Berridge et al., 2021; Wykes & Schueller, 2019) |
| Consumers should have control of their data (e.g., control over deletion or approving data input). | (Berridge et al., 2021; Branco et al., 2021; Köhler et al., 2022; Wykes & Schueller, 2019) |
| **Affordability** | **Supporting Articles** |
| Strive for an accessible price point for device and services. | (Freiesleben et al., 2021; Howes et al., 2024; Jacklin et al., 2020; Müller et al., 2017; Tetley et al., 2017) |
| **Environmental sustainability** | **Supporting Articles** |
| Strive for an environmentally sustainable products and business model. | (Branco et al., 2021; Interreg North-West Europe Certification-D 2023) |
| **Educational and Support Scaffolding** | **Supporting Articles** |
| Build user confidence in device (i.e., in their own ability to use technology). | (Hall et al., 2019; Köhler et al., 2022) |
| Provide comprehensive support before, at, and after implementation of LTDs into care (i.e., support over time). | (Tetley et al., 2017) |
| **Stakeholder inclusion and Trust** | **Supporting Articles** |
| Needs to be trust in the device, its capabilities, data security, etc. | (Köhler et al., 2022; Wan et al., 2016; Wykes & Schueller, 2019) |
| Outline design and development process (e.g., who was involved). | (Wykes & Schueller, 2019) |
| Development should be user centered (e.g., participatory design, co-creation, empathic design, human centered design, etc.). | (Branco et al., 2021; Freiesleben et al., 2021; Neubauer et al., 2018; Niemeijer et al., 2010; Toso et al., 2023) |
| Diverse stakeholders, including target users, should be included in development. | (Dröes et al., 2019; Müller et al., 2010; Wykes & Schueller, 2019) |
| **Anticipating risks and harms** | **Supporting Articles** |
| Ensure the LTD is secure to prevent harm from malfunctions or hacking, minimize the potential for misuse, and is robust enough to withstand accidental interactions such as falls, spills, etc. | (Abbas & Michael, 2022; Howes et al., 2024; Interreg North-West Europe Certification-D 2023) |
| Locators introduce new burdens and risks to PwD and caregivers. | (Grigorovich & Kontos, 2020; Howes et al., 2022; Howes & Gastmans, 2021; White et al., 2010) |

**References**

Abbas R, Michael K. Co-designing location-based services for individuals living with dementia: an overview of present and future modes of operation. IEEE Technol Soc Mag. 2022;41(2):42-46. [doi: 10.1109/MTS.2022.3173353]

Bantry-White E. Supporting ethical use of electronic monitoring for people living with dementia: social work's role in assessment, decision-making, and review. J Gerontol Soc Work. 2018;61(3):261-279. [doi: 10.1080/01634372.2018.1433738]

Bastoni S, Wrede C, da Silva MC, et al. Factors influencing implementation of eHealth technologies to support informal dementia care: umbrella review. JMIR Aging. 2021;4(4):e30841. [doi: 10.2196/30841]

Berridge C, Demiris G, Kaye J. Domain experts on dementia-care technologies: mitigating risk in design and implementation. Sci Eng Ethics. 2021;27(1):14.

Branco RM, Hendriks N, Lenaerts L, Wilkinson A. The challenges of creating design requirements for products for people with dementia. In: Brankaert R, Raber C, Houben M, Malcolm P, Hannan J, editors. Dementia Lab 2021: Supporting Ability Through Design. Springer; 2021:15-25. [doi: 10.1007/978-3-030-70293-9_2]

Brittain K, Degnen C, Gibson G, Dickinson C, Robinson L. When walking becomes wandering: representing the fear of the fourth age. Sociol Health Illn. 2017;39(2):270-284. [doi: 10.1111/1467-9566.12505]

Cooper J, Burrow S, Pusey H. What are the perceptions of people living with dementia, family carers, professionals and other potential stakeholders to the use of global positioning systems to promote safer outdoor walking?: a qualitative literature review. Disabil Rehabil Assist Technol. 2019;16(6):614-623. [doi: 10.1080/17483107.2019.1686074]

Dröes RM, Vermeer Y, Libert S, et al. Best practice guidance: human interaction with technology in dementia: recommendations based on the research conducted in the Marie Sklodowska Curie International Training Network INDUCT. INDUCT. 2019. URL: https://www.dementiainduct.eu/guidance/ [Accessed 2026-06-10]

Felber NA, Lipworth W, Tian YJA, Roulet Schwab D, Wangmo T. Informing existing technology acceptance models: a qualitative study with older persons and caregivers. Eur J Ageing. 2024;21(1):12. [doi: 10.1007/s10433-024-00801-5]

Freiesleben SD, Megges H, Herrmann C, Wessel L, Peters O. Overcoming barriers to the adoption of locating technologies in dementia care: a multi-stakeholder focus group study. BMC Geriatr. 2021;21(1):378. [doi: 10.1186/s12877-021-02323-6]

Gibson G, Newton L, Pritchard G, Finch T, Brittain K, Robinson L. The provision of assistive technology products and services for people with dementia in the United Kingdom. Dementia (London). 2016;15(4):681-701. [doi: 10.1177/1471301214532643]

Gibson G, Dickinson C, Brittain K, Robinson L. Personalisation, customisation and bricolage: how people with dementia and their families make assistive technology work for them. Ageing Soc. 2019;39(11):2502-2519. [doi: 10.1017/S0144686X18000661]

Grigorovich A, Kontos P. Towards responsible implementation of monitoring technologies in institutional care. Gerontologist. 2020;60(7):1194-1201. [doi: 10.1093/geront/gnz190]

Gullslett MK, Nilsen ER, Dugstad J. Next of kin's experiences with and attitudes towards digital monitoring technology for ageing people with dementia in residential care facilities. A qualitative study based on the voices of next of kin and care providers. Scand J Caring Sci. 2021;36:1094-1103. [doi: 10.1111/scs.13009]

Hall A, Wilson CB, Stanmore E, Todd C. Implementing monitoring technologies in care homes for people with dementia: a qualitative exploration using Normalization Process Theory. Int J Nurs Stud. 2017;72:60-70. [doi: 10.1016/j.ijnurstu.2017.04.008]

Hall A, Brown Wilson C, Stanmore E, Todd C. Moving beyond "safety" versus "autonomy": a qualitative exploration of the ethics of using monitoring technologies in long-term dementia care. BMC Geriatr. 2019;19(1):145. [doi: 10.1186/s12877-019-1155-6]

Hendriks N, Truyen F, Duval E. Designing with dementia: guidelines for participatory design together with persons with dementia. Presented at: Human-Computer Interaction—INTERACT 2013, 14th IFIP TC 13 International Conference; Sep 2-6, 2013; Cape Town, South Africa.

Howes J, Gastmans C. Electronic tracking devices in dementia care: a systematic review of argument-based ethics literature. Arch Gerontol Geriatr. 2021;95:104419. [doi: 10.1016/j.archger.2021.104419]

Howes J, Denier Y, Gastmans C. Electronic tracking devices for people with dementia: content analysis of company websites. JMIR Aging. 2022;5(4):e38865. [doi: 10.2196/38865]

Howes J, Denier Y, Vandemeulebroucke T, Gastmans C. The ethics of electronic tracking devices in dementia care: an interview study with developers. Sci Eng Ethics. 2024;30(3):17. [doi: 10.1007/s11948-024-00478-0]

Interreg North-West Europe Certification-D. Dementia friendly guidelines for SMEs. 2023. URL: https://vb.nweurope.eu/projects/project-search/certification-d-certification-of-technological-products-for-people-with-dementia-to-support-smes-in-innovation-and-business-growth/news/dementia-friendly-guidelines-for-smes/ [Accessed 2026-06-10]

Jacklin K, Pitawanakwat K, Blind M, Lemieux AM, Sobol A, Warry W. Peace of mind: a community-industry-academic partnership to adapt dementia technology for Anishinaabe communities on Manitoulin Island. J Rehabil Assist Technol Eng. 2020;7:2055668320958327. [doi: 10.1177/2055668320958327]

Köhler S, Görß D, Kowe A, Teipel SJ. Matching values to technology: a value sensitive design approach to identify values and use cases of an assistive system for people with dementia in institutional care. Ethics Inf Technol. 2022;24(3):27. [doi: 10.1007/s10676-022-09656-9]

Landau R, Werner S. Ethical aspects of using GPS for tracking people with dementia: recommendations for practice. Int Psychogeriatr. 2012;24(3):358-366. [doi: 10.1017/S1041610211001888]

Landau R, Werner S, Auslander GK, Shoval N, Heinik J. Attitudes of family and professional care-givers towards the use of GPS for tracking patients with dementia: an exploratory study. Br J Soc Work. 2009;39(4):670-692.

Landau R, Auslander GK, Werner S, Shoval N, Heinik J. Families' and professional caregivers' views of using advanced technology to track people with dementia. Qual Health Res. 2010;20(3):409-419. [doi: 10.1177/1049732309359171]

Landau R, Auslander GK, Werner S, Shoval N, Heinik J. Who should make the decision on the use of GPS for people with dementia? Aging Ment Health. 2011;15(1):78-84. [doi: 10.1080/13607861003713166]

Liu L, Daum C, Miguel Cruz A, Neubauer N, Perez H, Ríos Rincón A. Ageing, technology, and health: advancing the concepts of autonomy and independence. Healthc Manage Forum. 2022;35(5):296-300. [doi: 10.1177/08404704221110734]

Mahoney EL, Mahoney DF. Acceptance of wearable technology by people with Alzheimer's disease: issues and accommodations. Am J Alzheimers Dis Other Demen. 2010;25(6):527-531. [doi: 10.1177/1533317510376944]

Møller AK. The design of electronic tagging and tracking solutions to improve the safety and person-centered care for people with dementia. In: Gao Q, Zhou J, editors. Human Aspects of IT for the Aged Population: Healthy and Active Aging. Springer; 2020. [doi: 10.1007/978-3-030-50249-2_11]

Monville M, Schlögl S, Weichelt R, Windbichler R. Perspectives on technology use in dementia care—an exploratory study of nursing homes in Luxembourg. In: ICT for Health, Accessibility and Wellbeing. Springer; 2022. [doi: 10.1007/978-3-031-29548-5_5]

Müller C, Wan L, Hrg D. Dealing with wandering: a case study on caregivers' attitudes towards privacy and autonomy when reflecting the use of LBS. Presented at: GROUP 2010, ACM International Conference on Supporting Group Work; Nov 6-10, 2010; Sanibel Island, FL. [doi: 10.1145/1880071.1880082]

Müller I, Mertin M, Rolf M. Technology as an area of conflict between autonomy and safety-acceptance and attitudes of family caregivers in regard to technical assistance to ensure safe areas of movement for people with dementia diseases. Presented at: Proceedings of ICT4AWE 2017. 2017. URL: https://www.scitepress.org/Papers/2017/62830/

Neubauer NA, Liu L. Development and validation of a conceptual model and strategy adoption guidelines for persons with dementia at risk of getting lost. Dementia (London). 2021;20(2):534-555. [doi: 10.1177/1471301219898350]

Neubauer N, Hillier LM, Conway C, Beleno R, Liu L. Reflections of the use of locating technologies with persons with dementia: proceedings of a key stakeholder forum. Neurodegener Dis Manag. 2018;8(3):195-205. [doi: 10.2217/nmt-2018-0002]

Niemeijer A, Hertogh C. Implantable tags: don't close the door for Aunt Millie! Am J Bioeth. 2008;8(8):50-52.

Niemeijer AR, Frederiks BJ, Riphagen II, Legemaate J, Eefsting JA, Hertogh CM. Ethical and practical concerns of surveillance technologies in residential care for people with dementia or intellectual disabilities: an overview of the literature. Int Psychogeriatr. 2010;22(7):1129-1142. [doi: 10.1017/S1041610210000037]

Niemeijer AR, Frederiks BJM, Depla M, Legemaate J, Eefsting JA, Hertogh C. The ideal application of surveillance technology in residential care for people with dementia. J Med Ethics. 2011;37(5):303-310. [doi: 10.1136/jme.2010.040774]

Niemeijer AR, Depla MFIA, Frederiks BJM, Hertogh CMPM. The experiences of people with dementia and intellectual disabilities with surveillance technologies in residential care. Nurs Ethics. 2015;22(3):307-320. [doi: 10.1177/0969733014533237]

Nordgren A. How to respond to resistiveness towards assistive technologies among persons with dementia. Med Health Care Philos. 2018;21(3):411-421. [doi: 10.1007/s11019-017-9816-8]

Olsson A, Engström M, Lampic C, Skovdahl K. A passive positioning alarm used by persons with dementia and their spouses—a qualitative intervention study. BMC Geriatr. 2013;13:11. [doi: 10.1186/1471-2318-13-11]

Remmers H. Environments for ageing, assistive technology and self-determination: ethical perspectives. Inform Health Soc Care. 2010;35(3-4):200-210.

Robinson L, Brittain K, Lindsay S, Jackson D, Olivier P. Keeping In Touch Everyday (KITE) project: developing assistive technologies with people with dementia and their carers to promote independence. Int Psychogeriatr. 2009;21(3):494-502. [doi: 10.1017/S1041610209008448]

Spilker HS, Norby MK. Understanding the role of technology in care: the implementation of GPS-technology in dementia treatment. Ageing Int. 2019;44(3):283-299. [doi: 10.1007/s12126-018-9340-z]

Sundgren S, Stolt M, Suhonen R. Ethical issues related to the use of gerontechnology in older people care: a scoping review. Nurs Ethics. 2019;27(1):88-103. [doi: 10.1177/0969733019845132]

Tetley J, Koivunen E, Davenport D, et al. Dementia wristband report. Manchester Metropolitan University; 2017. URL: https://e-space.mmu.ac.uk/619326/

Toso F, Brankaert R, Hendriks N, Lenaerts L, Wilkinson A. Reflecting on living labs as multi-stakeholder collaborative networks to evaluate technological products for people living with dementia. Int J Environ Res Public Health. 2023;20(3):1796. [doi: 10.3390/ijerph20031796]

Wan L, Müller C, Randall D, Wulf V. Design of a GPS monitoring system for dementia care and its challenges in academia-industry project. ACM Trans Comput-Hum Interact. 2016;23(5):1-36. [doi: 10.1145/2963095]

Wan L. Dealing with wandering in dementia care: a developmental story of designing a GPS monitoring system and its challenges in a wider context [dissertation]. University of Siegen; 2017.

Welsh S, Hassiotis A, O'Mahoney G, Deahl M. Big brother is watching you—the ethical implications of electronic surveillance measures in the elderly with dementia and in adults with learning difficulties. Aging Ment Health. 2003;7(5):372-375.

Wherton J, Greenhalgh T, Procter R, Shaw S, Shaw J. Wandering as a sociomaterial practice: extending the theorization of GPS tracking in cognitive impairment. Qual Health Res. 2019;29(3):328-344. [doi: 10.1177/1049732318798358]

White EB, Montgomery P. Electronic tracking for people with dementia: an exploratory study of the ethical issues experienced by carers in making decisions about usage. Dementia (London). 2014;13(2):216-232. [doi: 10.1177/1471301212460445]

White EB, Montgomery P, McShane R. Electronic tracking for people with dementia who get lost outside the home: a study of the experience of familial carers. Br J Occup Ther. 2010;73(4):152-159.

Williamson B, Aplin T, de Jonge D, Goyne M. Tracking down a solution: exploring the acceptability and value of wearable GPS devices for older persons, individuals with a disability and their support persons. Disabil Rehabil Assist Technol. 2017;12(8):822-831. [doi: 10.1080/17483107.2016.1272140]

Wrede C, Braakman-Jansen A, van Gemert-Pijnen L. Requirements for unobtrusive monitoring to support home-based dementia care: qualitative study among formal and informal caregivers. JMIR Aging. 2021;4(2):e26875. [doi: 10.2196/26875]

Wykes T, Schueller S. Why reviewing apps is not enough: transparency for trust (T4T) principles of responsible health app marketplaces. J Med Internet Res. 2019;21(5):e12390. [doi: 10.2196/12390]

Yang YT, Kels CG. Does the shoe fit? Ethical, legal, and policy considerations of global positioning system shoes for individuals with Alzheimer's disease. J Am Geriatr Soc. 2016;64(8):1708-1715. [doi: 10.1111/jgs.14223]
